# Ionotropic Glutamate Receptor AMPA 1 Is Associated with Ovulation Rate

**DOI:** 10.1371/journal.pone.0013817

**Published:** 2010-11-03

**Authors:** Mayumi Sugimoto, Shinji Sasaki, Toshio Watanabe, Shota Nishimura, Atsushi Ideta, Maya Yamazaki, Keiko Matsuda, Michisuke Yuzaki, Kenji Sakimura, Yoshito Aoyagi, Yoshikazu Sugimoto

**Affiliations:** 1 National Livestock Breeding Center, Nishigo, Japan; 2 Shirakawa Institute of Animal Genetics, Nishigo, Japan; 3 Embryo Transfer Center ZEN-NOH, Kamishihoro, Japan; 4 Brain Research Institute, Niigata University, Niigata, Japan; 5 Department of Physiology, Keio University School of Medicine, Tokyo, Japan; Pennsylvania State University, United States of America

## Abstract

Ionotropic glutamate receptors mediate most excitatory neurotransmission in the central nervous system by opening ion channels upon the binding of glutamate. Despite the essential roles of glutamate in the control of reproduction and anterior pituitary hormone secretion, there is a limited understanding of how glutamate receptors control ovulation. Here we reveal the function of the ionotropic glutamate receptor AMPA-1 (GRIA1) in ovulation. Based on a genome-wide association study in *Bos taurus*, we found that ovulation rate is influenced by a variation in the N-terminal leucine/isoleucine/valine-binding protein (LIVBP) domain of GRIA1, in which serine is replaced by asparagine. GRIA1^Asn^ has a weaker affinity to glutamate than GRIA1^Ser^, both in *Xenopus* oocytes and in the membrane fraction of bovine brain. This single amino acid substitution leads to the decreased release of gonadotropin-releasing hormone (GnRH) in immortalized hypothalamic GT1-7 cells. Cows with GRIA1^Asn^ have a slower luteinizing hormone (LH) surge than cows with GRIA1^Ser^. In addition, cows with GRIA1^Asn^ possess fewer immature ovarian follicles before superovulation and have a lower response to hormone treatment than cows with GRIA1^Ser^. Our work identified that GRIA1 is a critical mediator of ovulation and that GRIA1 might be a useful target for reproductive therapy.

## Introduction

Cattle, like humans, usually have single ovulations. Superovulation is a method often used in the livestock industry to obtain more descendants from exceptional dams, but the treatments produce inconsistent results [1]. Identifying the gene variants responsible for the variable responses to superovulation treatments may help toward selecting cows that will be more responsive to the superovulation treatment. The molecular mechanisms that regulate ovarian follicular development in mammals are poorly understood, mainly because of species-specific differences between mono-ovulatory and polyovulatory animals. Analyses of mouse models with female fertility defects have revealed numerous gene products with key roles at various stages of ovarian folliculogenesis [2]. It is important, however, to identify specific gene products that control ovulation in mono-ovulatory animals.

**Figure 1:**
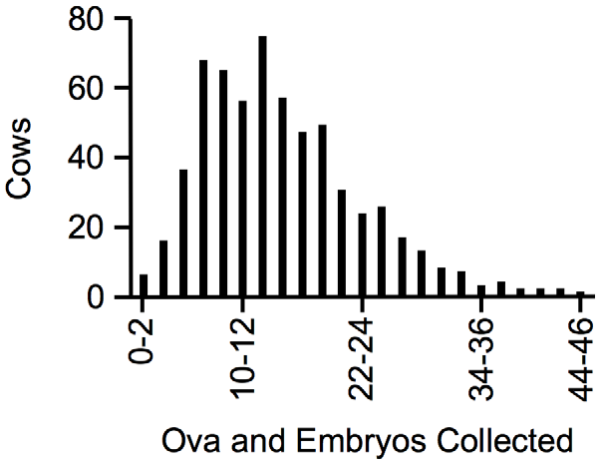
The average number of ova and embryos collected per superovulation over five treatments.

Although genome-wide scans to identify the genes that influence ovulation rate in cattle have been performed, no specific genes have been identified to date [3]. The estimated maternal heritability of ovulation rate is relatively high (0.23) [4], but other factors, such as environment and technical expertise, are potential impediments to determining genetic variations and identifying the specific genes involved. To overcome this problem, we collected blood and superovulation records of 639 Japanese Black cattle at the Embryo Transfer Center ZEN-NOH, where professionals routinely perform repeated superovulation treatments to collect embryos for commercial purposes. The average number of ova and embryos collected over five treatments ranged from 0.4 to 44.2 per superovulation with a median of 13.8 (Figure 1).

Using these samples, we found that ovulation rate in cattle was associated with the GRIA1. GRIA1 mediates most excitatory neurotransmission by opening ion channels upon the binding of glutamate [5], Cows with GRIA1^Asn^, in which serine is replaced by asparagine, have fewer immature follicles in their ovaries than cows with GRIA1^Ser^. This single amino acid substitution decreases the affinity of the receptor to glutamate. Immortalized hypothalamic cells transfected with GRIA1^Asn^ release less GnRH than do cells with GRIA1^Ser^. Cows with GRIA1^Asn^ have a slower LH surge than cows with GRIA1^Ser^. Our work would lead to more efficient breeding practices for cattle and to a better understanding of folliculogenesis in mono-ovulatory animals.

## Results

### GRIA1 is associated with ovulation rate in cattle

Among the samples we collected at the Embryo Transfer Center ZEN-NOH, we selected 42 cows from which many ova and embryos were collected (high, ≥18.2) and 42 cows from which only a few ova and embryos were collected (low, ≤9.6). To reduce the effects of specific sires, fewer than five cows derived from the same father were included. Based on typing 1154 microsatellite markers covering from chromosomes 1 to 29 and X, the population structure of the selected samples was evaluated with STRUCTURE [6] and we found no evidence of a systematic bias (Figure S1). The stratification [7] of our samples was also low (λ = 1.104). The estimated effective population size [8] of the selected samples, 29.6, was similar to previously reported population sizes of Japanese Black cattle [9] (14.0–52.1), indicating that the sample in this study well represented the Japanese Black cattle population.

**Figure 2:**
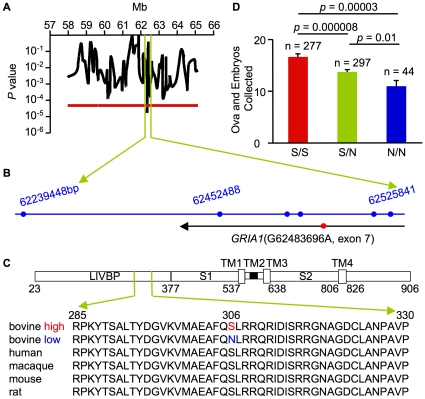
GRIA1 is associated with ovulation rate in cattle. (A) Association signals with ovulation rate on chromosome 7 using plots of the *P* values for Fisher's exact test after estimating haplotypes of consecutive marker pairs by expectation-maximization algorithm. The red line represents the threshold for genome-wise significance after correction using the Bonferroni correction for multiple comparisons. (B) The map of markers (blue circles) and a gene (black line) found in the candidate region of chromosome 7. The red circle represents an SNP found in the gene. (C) Schematic illustration of GRIA1 that harbors 4 transmembrane (TM) domains and the LIVBP domain, which includes alignment of partial mammalian amino acid sequences. (D) The average number of ova and embryos collected per superovulation of S/S, S/N, and N/N cows over five treatments. Data are presented as mean ± SEM. *P* values were calculated by Student's t-test.

We scanned a total of 84 bovine genomes and revealed a significant association at the chromosome-wise or genome-wise level between ovulation rate and markers associated with chromosomes 2, 3, 4, 6, 7, 8, 9, 10, 13, 14, 15, 16, 19, 21, 23, 24, 27, and X (Figure S2, Panel A). Minor haplotypes whose frequencies were less than 5 % were removed from the calculations and all of the markers were reasonably within Hardy-Weinberg equilibrium (HWE, *P*>0.002 for either high or low). When the number of samples was increased from 84 to 134 (67 high ≥16.6, 67 low ≤7.7), the analysis demonstrated that chromosomes 6, 7, 8, 14, 16, 19, and 24 maintained a significant association (Figure S2, Panel B). Further analysis with an additional 50 markers showed the most robust association on chromosome 7 (Figure S2, Panel C). Scanning of this chromosome with an additional 86 markers indicated that candidate genes were located in the region between 62 and 63 Mb (Figure 2, Panel A), which harbors *GRIA1* (Figure 2, Panel B).

To detect possible causative polymorphisms in *GRIA1*, we sequenced all exons of this gene in selected samples with homozygous high- or low-specific haplotypes (Figure S3) and found that the high samples had G alleles, while the low samples had A alleles in exon 7 (Figure 2, Panel B). The identified single nucleotide polymorphism (SNP) and its three neighboring microsatellite markers were in strong linkage disequilibrium (LD) with each other; pairwise χ^2′^ measures were all greater than 0.7 (Figure S4). This SNP replaces a serine with an asparagine at amino acid residue 306 (S306N) in the LIVBP domain of GRIA1, which is highly conserved from rat to human (Figure 2, Panel C). Encouraged by this finding, we sequenced *GRIA1* in all 639 samples and confirmed that S306N of GRIA1 is linked to ovulation rate and one copy of GRIA1^Asn^ decreases the number of ova and embryos collected per superovulation by three ova (Figure 2, Panel D).

To check the general ratio of this naturally occurring variant in cattle, we sequenced *GRIA1* in commercially available sires and observed that the Mendelian 1∶2∶1 genotype ratio remained and this SNP does not depart from HWE in either the Japanese Black (*P* = 0.125) or Holstein populations (*P* = 0.547, Figure S5, Panel A). Intensive genetic selection for high yields of meat or milk among beef or dairy cattle has not changed the frequency of variants in *GRIA1*. On the other hand, in the Embryo Transfer Center ZEN-NOH, they choose cows to collect many embryos after conducting three to five superovulations, which might reduce the number of N/N cows in their population (44/618 = 7.1%; HWE, *P* = 0.004, Figure 2, Panel D). We did not have enough superovulation records to confirm that GRIA1 influences ovulation rate among Holsteins; however, the average conception rate of the N/N population was lower than the S/S population based on artificial insemination (AI) records (Figure S5, Panel B). GRIA1 might affect the conception rate through effects on the ovulation rate in cattle.

### S306N in GRIA1 affects ligand affinity

The functions of the N-terminal LIVBP domain of GRIA1 are largely unknown. A 170-residue deletion in the LIVBP domain of the ionotropic glutamate receptor delta 2 (Grid2) impairs its exit from the endoplasmic reticulum in mice [10]. To examine whether S306N in GRIA1 affects protein transport to the cell surface, we stained GRIA1-expressing HEK 293 cells with an anti-hemagglutinin (HA) antibody under nonpermeabilizing conditions. There was no difference between GRIA1^Ser^, GRIA1^Asn^, and murine wild-type Grid2 as a positive control (PC) in the fluorescence intensity ratio of the HA staining to green fluorescent protein (GFP) expressed in the nucleus (Figure S6, Panel A). Expression of murine deletion mutant Grid2 as a negative control (NC) under nonpermeabilizing and permeabilizing conditions confirmed the plausibility of the experiments (Figure S6, Panel B). We also compared GRIA1 expression in membrane fractions from the brain of S/S, S/N, and N/N cows and found no difference (Figure S6, Panel C). A single mutation, not a large deletion, of the LIVBP domain might not affect quality control mechanisms.

**Figure 3:**
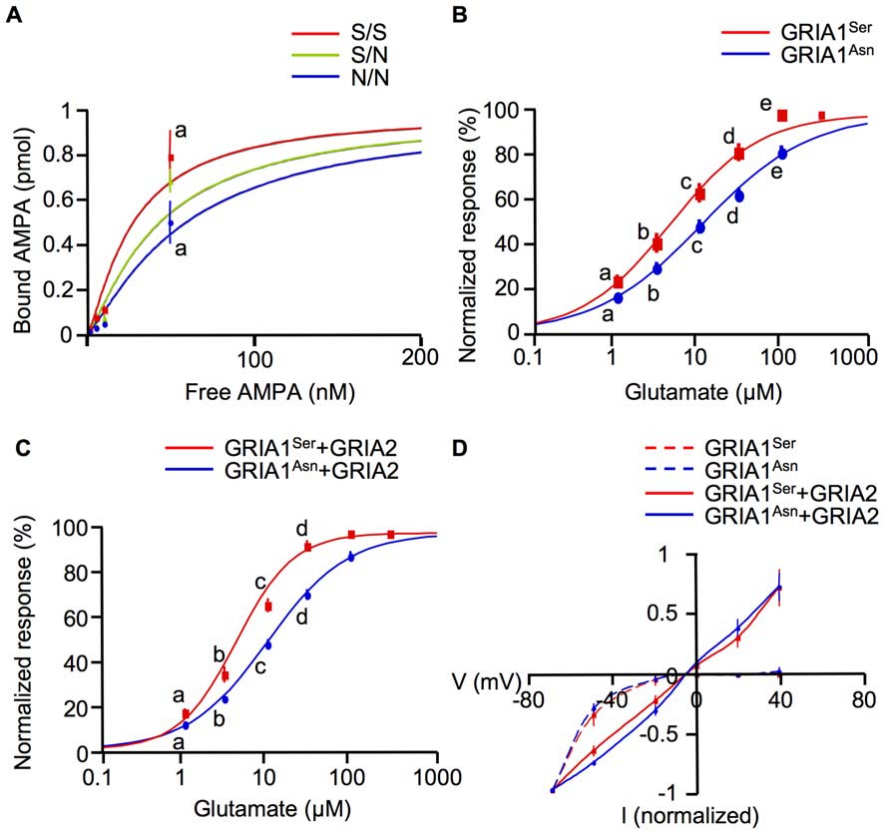
S306N in GRIA1 affects ligand affinity. (A) Saturation curves for [^3^H] AMPA binding to brain of S/S (red, n = 3), S/N (green, n = 3), and N/N cows (blue, n = 3). Data are presented as mean ± SEM. a indicates *p*<0.05 by Student's t-test between S/S and N/N cows. (B) Dose-response curves of *Xenopus* oocytes expressing GRIA1^Ser^ (red, n = 10) or GRIA1^Asn^ (blue, n = 9). Normalized response means current normalized to the current evoked by a saturating dose of glutamate. Data are presented as mean ± SEM. a–e indicate *p*<0.05 by Student's t-test between GRIA1^Ser^ and GRIA1^Asn^. (C) Dose-response curves of *Xenopu*s oocytes expressing GRIA1^Ser^ (red, n = 8) or GRIA1^Asn^ (blue, n = 8) with GRIA2. Data are presented as mean ± SEM. a–d indicate *p*<0.05 by Student's t-test between GRIA1^Ser^ and GRIA1^Asn^. (D) *I–V* relationships of oocytes expressing GRIA1^Ser^ (red broken line; n = 4), GRIA1^Ser^ with GRIA2 (red line; n = 5), GRIA1^Asn^ (blue broken line; n = 4), and GRIA1^Asn^ with GRIA2 (blue line; n = 5). Each *I–V* relationship was normalized to the current obtained at −70 mV. Data are presented as mean ± SEM.

The N-terminal domain of ionotropic glutamate receptors mediates dimerization [11] and affects ligand affinity [12]. GRIA1^Asn^ might have lower ligand affinity than GRIA1^Ser^. Indeed, a ligand binding assay using membrane fractions extracted from bovine brain showed that the K_D_ for AMPA, was 27.7, 43.6, and 59.6 nM in S/S, S/N, and N/N cows, respectively (Figure 3, Panel A). Moreover, current responses in *Xenopus* oocytes injected with *GRIA1* mRNA revealed that the EC_50_ for GRIA1^Ser^ and GRIA1^Asn^ was 4.5 and 10.7 µM, respectively (Figure 3, Panel B). When coexpressed with GRIA2, the dimer partner of GRIA1, the EC_50_ for GRIA1^Ser^ (4.9 µM) was also lower than that for GRIA1^Asn^ (10.4 µM, Figure 3, Panel C), although the *I–V* relationships of both GRIA1 were similar (Figure 3, Panel D). The N-terminal domain of *N*-methyl-_D_-aspartate receptors controls channel gating [13]. S306N in the N-terminal LIVBP domain of GRIA1 might affect its ligand affinity through controlling dimer assembly.

### S306N in GRIA1 affects hormone release

**Figure 4:**
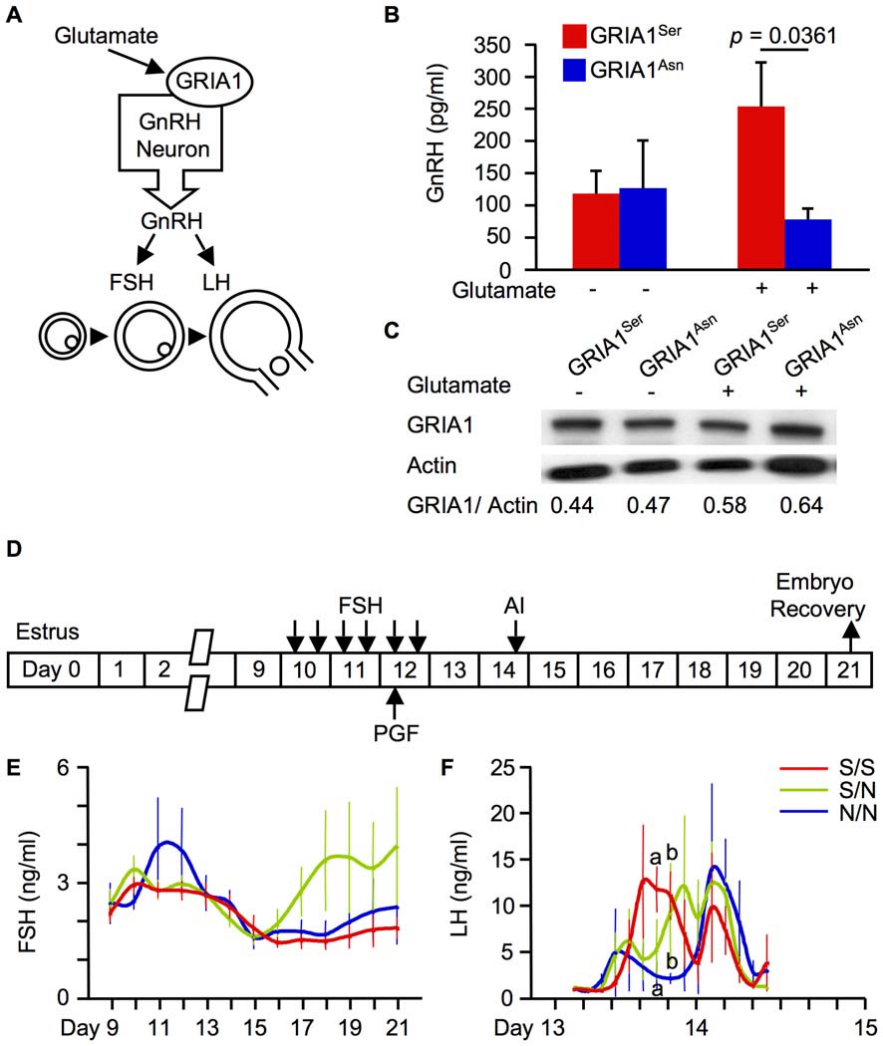
S306N in GRIA1 affects hormone release. (A) Schematic of ovulation control. (B) The concentration of GnRH released from GT1-7 cells expressing GRIA1^Ser^ (red) or GRIA1^Asn^ (blue). Data are presented as mean ± SEM (n = 3 each). *P* values were calculated by Student's t-test. (C) Representative immunoblots with anti-HA (GRIA1) and anti-actin (control) antibody. (D) The protocol for superovulation in cattle. (E–F) The concentration of FSH (E) and LH (F) in the serum of S/S (red, n = 6), S/N (green, n = 5), and N/N cows (blue, n = 4) during superovulation. Data are presented as mean ± SEM. a and b indicate *p*<0.05 by Student's t-test between S/S and N/N cows.

The molecular link between GRIA1 and ovulation is not well understood. To address this issue, we first investigated GnRH secretion in murine immortalized hypothalamic GT1-7 cells [14] expressing GRIA1, because glutamate stimulates hypothalamic GnRH release [15] (Figure 4, Panel A). As expected, stimulation of GT1-7 cells expressing GRIA1^Ser^ with 100 µM glutamate for 30 min induced the release of more GnRH than that from cells expressing GRIA1^Asn^ (Figure 4, Panel B). There was no difference in the transfection efficiency between GRIA1^Ser^ and GRIA1^Asn^ (Figure 4, Panel C). S306N in GRIA1 might influence GnRH release by altering glutamate affinity.

GnRH regulates follicle stimulating hormone (FSH) and LH secretion in the anterior pituitary [16] (Figure 4, Panel A). FSH induces follicle growth [17] while LH induces ovulation [18]. Because GRIA1 variants might influence FSH and LH secretion through the regulation of GnRH release, we examined FSH and LH concentrations in the serum of S/S, S/N, and N/N cows during superovulation. For superovulation, cattle were administered decreasing doses of FSH twice a day on Days 10, 11, and 12 of their estrus cycle (Figure 4, Panel D). The prostaglandin (PG) F_2α_ was also administered at the fifth FSH treatment. We detected no differences in the FSH concentration between S/S and N/N cows (Figure 4, Panel E). On the other hand, N/N cows exhibited slower LH surge than S/S cows at 55 h after PGF_2α_ treatment (Figure 4, Panel F and Figure S7).

### S306N in GRIA1 affects ovulation

**Figure 5:**
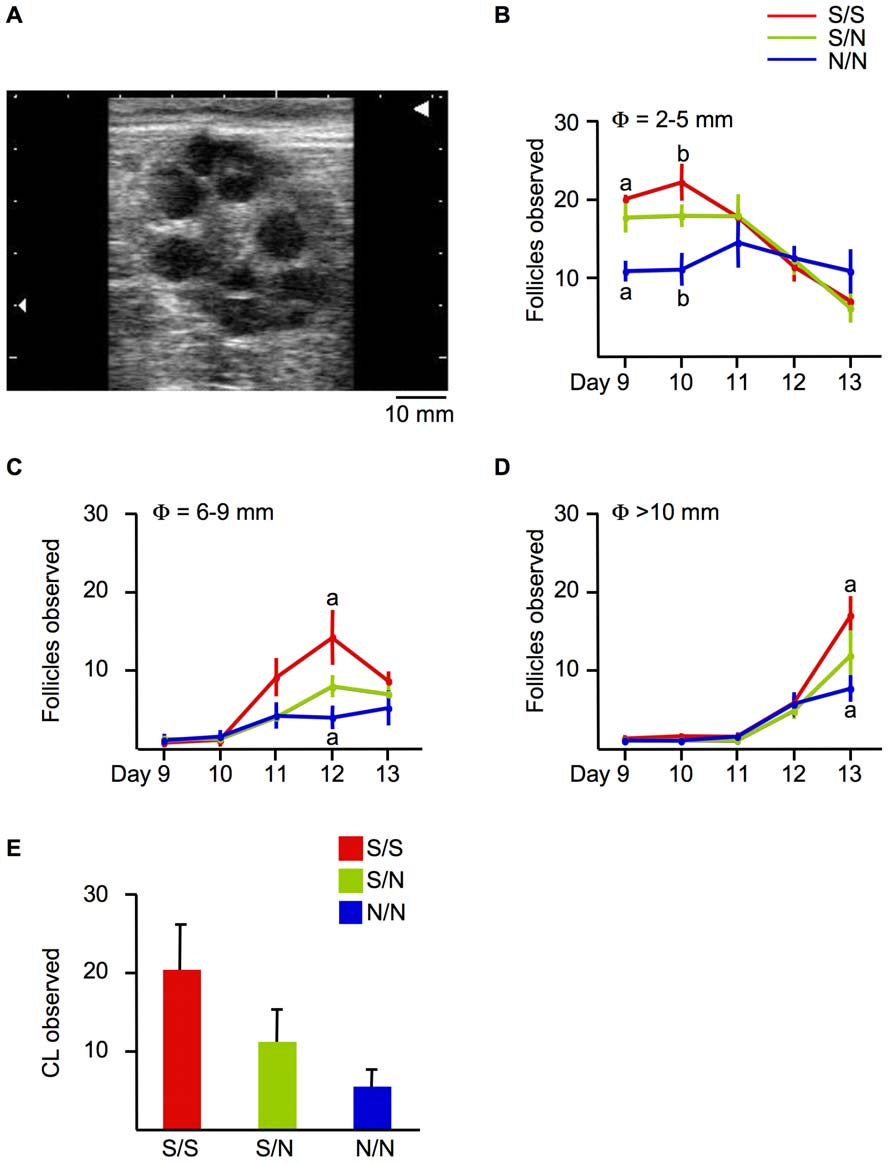
S306N in GRIA1 affects ovulation. (A) Representative photo of an ovary of an S/S cow at Day 13 by ultrasound scanning. Mature follicles (φ>10 mm) were recognizable. (B–D) The average number of immature follicles (B, φ = 2–5 mm), maturing follicles (C, φ = 6–9 mm), and mature follicles (D, φ>10 mm) observed by ultrasound scanning of S/S (red, n = 6), S/N (green, n = 5), and N/N cows (blue, n = 4) during superovulation. Data are presented as mean ± SEM. a and b indicate *p*<0.05 by Student's t-test between S/S and N/N cows. (E) The average number of CL of S/S (red, n = 6), S/N (green, n = 5), and N/N cows (blue, n = 4) after superovulation. Data are presented as mean ± SEM.

To more closely observe the effects of S306N in GRIA1 on folliculogenesis during superovulation, we examined the ovaries in S/S, S/N, and N/N cows by ultrasound scanning (Figure 5, Panel A). N/N cows had fewer immature follicles (φ = 2–5 mm) than S/S cows before superovulation at Day 9 (Figure 5, Panel B). Moreover, N/N cows also had fewer maturing follicles (φ = 6–9 mm) than S/S cows and the ratio of maturing follicles to total follicles in N/N cows was lower than that of S/S cows at Day 12 (Figure 5, Panel C, 16.5±6.4 % vs. 42.9±5.7 %, *P* = 0.0097), indicating that N/N cows responded less to FSH administration than S/S cows. At Day 13, N/N cows had fewer mature follicles (φ>10 mm) than S/S cows (Figure 5, Panel D). We also counted corpora lutea (CL), however, it was not a statistically significant difference between S/S and N/N cows (*P* = 0.07, Figure 5, Panel E).

## Discussion

We identified a naturally occurring genetic variant with a rather large effect on a quantitative trait, ovulation rate. Based on typing many microsatellite markers, we narrowed the region associated with ovulation rate, where locates only one candidate gene, *GRIA1*. GRIA1 carries one SNP changing from serine to asparagine, Cows with GRIA1^Ser^ have an average of six more ova and embryos than cows with GRIA1^Asn^, comprising a 10% variation of the average number of ova and embryos collected during five superovulations. Our results might provide a method for selecting cows to be superovulated so that ova and embryos can be collected more efficiently.

Based on *in vitro* stiudies, we found that this single amino acid substitution changes the affinity of the receptor to glutamate. Immortalized hypothalamic cells transfected with GRIA1^Asn^ release less GnRH than do cells with GRIA1^Ser^. Based on *in vivo* studies, we observed that cows with GRIA1^Asn^ have a slower LH surge than cows with GRIA1^Ser^ at 55 h after PGF_2α_ treatment, although cows usually exhibit LH surge at 43.9 ± 1.5 h (mean ± SEM, n = 28) after PGF_2_α treatment [19], LH administration from Day 7 to 13 stimulates continued growth of large follicles [20], suggesting that earlier LH surges might increase the number of follicles that are ovulated. Moreover, AMPA infusion into the third cerebral ventricle in ovariectomized estrogen-primed adult female rats induces LH release [21]. A dominant negative mutant of GRIA1 expressed in the preoptic area of female rats attenuates LH secretion [22]. Therefore, GRIA1 might control LH secretion by regulating GnRH release.

In addition to LH secretion, GRIA1 polymorphism might affect the ovary directly through GnRH. Choi et al. [23] observed expression of GnRH receptors in the human ovary during follicular development, Because hypothalamic cells with GRIA1^Ser^ release more GnRH than do cells with GRIA1^Asn^, released GnRH might contribute ovulation in the ovary directly.

Many studies have evaluated various physiologic aspects of Holstein cows that have high and low numbers of follicles. For example, intrafollicular concentration of estradiol was greater for cows with low antral follicle count than for cows with high antral follicle account [24]. GRIA1 might influence concentrations of other hormone in addition to LH.

In this study, we demonstrated that GRIA1 has an important role in ovarian follicular development among cattle, a mono-ovulatory animal. Although glutamate influences LH release [21], reproduction is not affected in mice deficient in GRIA1 [25]. Mice lacking Gria2 in GnRH neurons have normal fertility despite impaired reproductive behavior [26]. It might be difficult to observe the effects of GRIA1 in folliculogenesis in mice, a polyovulatory animal. In humans, a mono-ovulatory species, superovulation is often conducted for the treatment of infertility [27]. Increasing the number of ova and embryos released by superovulation combined with *in-vitro* fertilization to enhance conception improves pregnancy rates. Our results indicate that GRIA1 might be a useful target for reproductive therapy in women.

## Materials and Methods

### Ethics Statement

All animal experimentation was undertaken with the approval of the National Livestock Breeding Center Committee on Animal Research (H21-35).

### Superovulation

The cattle were administered 5 armour units (AU) of FSH (Antrin R-10, Kawasaki-Mitaka, Kanagawa, Japan) at 0800h and 1600h on Day 10, 3 AU at 0800 h and 1600 h on Day 11, and 2 AU at 0800 h and 1600 h on Day 12 of their estrus cycle. A PGF_2α_ analogue (3 ml Resipron-C containing 0.25 mg/ml cloprostenol, ASKA Pharmaceutical Co., Ltd., Tokyo, Japan) was administered at 0800 h on Day 12. The cattle were inseminated with one dose of frozen-thawed semen in the afternoon on Day 14 or on the morning of Day 15. Ova and embryos were recovered by uterine flushing on Day 21. Superovulated ovaries were examined by real-time B-mode ultrasonography (Tringa Linear Ultrasound System; Esaote-Pie Medical, Maastricht, Netherlands) with a 7.5 MHz rectal transducer.

### Mapping

Genomic DNA was isolated from the blood or semen using NA-1000/48S (Kurabo, Tokyo, Japan) or Easy-DNA kit (Invitrogen, Carlsbad, CA). Fluorescence-labeled (CA)n microsatellite markers were selected from the Shirakawa-USDA genetic map [28]. Genotyping was performed using the ABI 3730 sequencer and GeneMapper (Applied Biosystems, Foster City, CA).

The population structure of our samples was estimated with STRUCTURE [6]. 155 markers were extracted from 1154 markers, with at least a 20-cM interval. We set 100,000 Markov chain Monte Carlo interactions including 10,000 burn-in interactions, and assumed the subpopulation number to be 2. Eighty-four individuals were separated into populations 1 and 2.

The degree of stratification of the samples in this study was examined using the genomic control method [7]. Briefly, λ was the observed median of χ^2^ values of multiple testing divided by the expected median of χ^2^ value (P = 0.5) under the null hypothesis. λ indicates degree of inflation of χ^2^ statistic values throughout multiple tests. If there is no stratification, λ is equal to 1. Because the degree of freedom of each test in this study was not always the same (from 1 to 9 in 1122 tests), the overall average of λ weighted by the number of tests for each degree of freedom was calculated. χ^2^ values with Yates' correction for continuity were used because the expected value of the cells in the contingency tables was often less than 5.

Effective population size, Ne, was estimated as the coefficient in the equation reported by Sved [8];



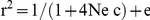



where r^2^ is the linkage disequilibrium coefficient, c is a genetic distance between two markers in Morgans, and e is the residual error. To estimate an effective population size using microsatellite markers, χ^2′^ value is preferred over r^2^ as the linkage disequilibrium coefficient [29]. Effective population size was estimated with χ^2′^ values of 5192 pairs of two markers on all autosomes within a 15-cM window. Non-linear regression of genetic distances on χ^2′^ values was performed with the nls function of R software (http://www.r-project.org/).

Fisher's exact test was used for association studies after estimating haplotypes of consecutive marker pairs by expectation-maximization algorithm as described previously [30]. Tests for deviation from Hardy-Weinberg equilibrium were computed as described previously [30].

All exons of bovine *GRIA1* were sequenced after amplifying by polymerase chain reaction (PCR) with primers shown on Table 1. A SNP in *GRIA1* was typed with primers GRIA1exon7 F and R (Table 1).

**Table 1:** Primer sequences.

Name	Sequence
GRIA1exon1 F	ATTGGACCTGGGCTTCTTTT
GRIA1exon1 R	CAGCACAACAGCCCATAGTG
GRIA1exon2 F	GCCCAGATACCATGTTGCAT
GRIA1exon2 R	CCCTCCCTTTAATGGTCGAT
GRIA1exon3 F	AATTTCTCTCTTCTCCTTTTCCA
GRIA1exon3 R	TTCAATGTCTCCCTCCCCTA
GRIA1exon4 F	TGTCCTACTCACCCCACCTC
GRIA1exon4 R	CCTTCGCTGCGCTCACTA
GRIA1exon5 F	GGATAGGACAGGGCAGTCAG
GRIA1exon5 R	AGTTGAGGGCTTGCAGTGTT
GRIA1exon6 F	ACTTCCTTCCCTTCCTCACC
GRIA1exon6 R	AGAGACCAGCAGGGTCCTTT
GRIA1exon7 F	AGCCTCCCTACCAGCTCTCT
GRIA1exon7 R	CGTTGTTGCCAGCCTCAC
GRIA1exon8 F	AGGAAAAGCCAGCAAATGAA
GRIA1exon8 R	TGGAAGCAAAGGGAGCTTTA
GRIA1exon9 F	CCACACCCCTCTCCTTAACA
GRIA1exon9 R	CCTCTGTGTTGGCCTAGCTC
GRIA1exon10 F	GTCTCCCATTTCTCCACCAG
GRIA1exon10 R	CCCTACCCTATGGGGGACT
GRIA1exon11 F	GGAACAGAGGGCTGATGAAC
GRIA1exon11 R	TGATGAGAAGAGCCGACTGA
GRIA1exon12 F	TGCTTCTGATATCTCTTCCCTTG
GRIA1exon12 R	TGGTTCCCTTCTGTGGAAGT
GRIA1exon13 F	CATTCCAGCTTTGTTCTGTCC
GRIA1exon13 R	GGAAGCAAATCTTGGCTACTTA
GRIA1exon14 F	TGCTGAGTGTTCTCCACCTG
GRIA1exon14 R	GGTTACTGGCCATGCTGTTT
GRIA1exon15 F	CCTGGCTCATTGGACTCTTC
GRIA1exon15 R	CCAGGAAACCCAGCTGTATC
GRIA1exon16 F	GCTGGGTAGGGAGGAGAGTT
GRIA1exon16 R	GCTCCAGTGACACAGGCTCT
GRIA1 F	GGAATTCTTCCCCAACAATATCCAG
GRIA1 R	TTTGTACAAGGCGGCCGCTTACAATCCAGTGGCTC
GRIA1InFusion F	CCGGGATCCCGAATTCGCCACCATGGTCCTGCTGGT
GRIA1InFusion R	CCTGAGGAGTGAATTCTTACAATCCAGTGGCTCCCA

### Immunohistochemical analysis

Bovine *GRIA1* coding sequences were derived by reverse-transcription PCR with primers GRIA1F and R (Table 1). These coding sequences were cloned into pTracer-nucGFP (Invitrogen) to express GRIA1 protein with a hemagglutinin (HA) tag in its N-terminal and GFP in the nucleus were transfected into HEK 293 cells, which were provided by the RIKEN CELL BANK (Tsukuba, Japan). Immunocytochemical analysis was performed as described previously [31]. Stained cells were visualized with a microscope (IX81, Olympus, Tokyo, Japan) equipped with a CCD camera (Luca, Andor Technology, Tokyo, Japan). The intensity of staining based on 10 cells each from 3 independent experiments was quantified by Andor iQ1.9 (Andor Technology).

### Western blotting analysis

Membrane fractions were extracted from bovine brain as described previously [32], and 25 µg of protein were resolved by SDS-PAGE and transferred to polyvinylidene difluoride membranes. The blots were incubated with rabbit polyclonal antibody to GRIA1 (1∶1000, AB1504, Millipore, Tokyo, Japan) or actin (1∶200, A2066, Sigma-Aldrich, St. Louis, MO).

### Ligand binding analysis

Protein (500 µg/ml) was used for a ligand binding assay with [^3^H] AMPA (392.2 GBq/mmol, 1–200 nM) as described previously [33]. Non-specific binding obtained by incubating 1 mM _L_- glutamate in the binding assay was subtracted from total binding to yield specific [^3^H] AMPA- binding.

### Electrophysiological analysis

Bovine *GRIA1* mRNAs were synthesized using mMESSAGE mMACHINE® SP6 Kit (Applied Biosystems) and injected into *Xenopus* oocytes as described previously [34]. Whole-cell current responses to 1, 3, 10, 30, 100, and 300 µM glutamate were recorded as described previously [35].

### GnRH enzyme immunoassay

We amplified bovine *GRIA1* that had been cloned into pTracer-nucGFP with primers GRIA1InFusionF and R and then switched into pCAGGS (N-R) [36] using the In-Fusion™ Advantage PCR Cloning Kit (Takara Bio Inc., Shiga, Japan) to produce HA-tagged protein under the control of a ubiquitous strong promoter that was based on the β-actin promoter.

GT1-7 cells were cultured in six-well dishes coated with poly-L-ornithine (BD Biocoat [PLO/LM] 354658, Becton Dickinson and Company, Franklin Lakes, NJ) with conditioned-Dulbecco's modified Eagles medium (C-MEM), which was comprised of a 1∶1 mixture of conditioned medium from mouse embryonic astrocytes in primary culture and Dulbecco's modified Eagles medium with high glucose (Invitrogen) supplemented with 10% fetal calf serum, penicillin (100 U/ml), and streptomycin (100 µg/ml). When cells reached 90 % confluence, C-MEM was replaced with Opti-MEM (Invitrogen) and transfected with Lipofectamine 2000 (Invitrogen) for 24 h.

The next day, confluent GT1-7 cells were treated as described previously [37]. The concentration of GnRH in the media was assayed using an LH-releasing hormone enzyme immunoassay kit (S-1217, Peninsula Laboratories, LLC, San Carlos, CA) according to the manufacturer's instructions.

Cells were extracted with phosphate-buffered saline containing 0.5 % sodium deoxycholate and 0.5 % NP-40. Aliquots of 5 µg of protein were used for Western blotting with rat monoclonal antibody to HA (1:1000, 11867423001, Roche, Mannheim, Germany).

### Radioimmunoassay

Blood was collected daily by coccygeal venipuncture from Day 9 to Day 21 and alternately once every 2 h from 30 to 58 h after PGF_2α_ treatment. Blood was stored at 4°C until serum was harvested by centrifugation. Serum was stored at −30°C until assayed. The serum concentrations were measured by radioimmunoassay for FSH [38] and LH [39]. Assay sensitivity of FSH and LH were 0.003 ng/tube and 0.008 ng/tube, respectively. Intra-assay coefficients of variation for FSH and LH were 2.7 % and 3.5 %, respectively.

## Supporting Information

Figure S1 The population structure of analyzed samples based on STRUCTURE. The population structure of our samples was estimated with STRUCTURE [6].155 markers were extracted from 1154 markers, with at least a 20-cM interval. We set 100,000 Markov chain Monte Carlo interactions including 10,000 burn-in interactions, and assumed the subpopulation number to be 2. Eighty-four individuals were separated into populations 1 and 2. The inferred proportion of ancestry in population 1 of high (red) and low (blue) were similar.
(0.06 MB TIF)

Figure S2 Chromosome 7 is associated with ovulation rate in cattle. (A–C) Association signals with ovulation rate using plots of the *P* values for Fisher's exact test after estimating the haplotypes of consecutive marker pairs by the expectation-maximization algorithm. Different bands of green are used to differentiate marker pairs on consecutive chromosomes. Blue and red lines represent the thresholds for chromosome-wise and genome-wise significance based on Bonferroni's correction for multiple comparisons, respectively. (A) Genome-wide scans. (B) Scans at selected chromosomes with additional samples. (C) Scans at selected chromosomes with additional markers.
(0.39 MB TIF)

Figure S3 Samples for sequencing are selected by their haplotypes. (A) Frequency of haplotypes of consecutive marker pairs in the candidate region. Red and blue indicate ‘high’- and ‘low’-specific haplotypes, respectively. NLBCMS3, 4, 5, 6, and 7 and BMS792 are microsatellite markers located in the critical region. (B) Selected samples for sequencing. Sample 1 and 2 represent ‘high’ samples which have both ‘high’-specific haplotypes and ‘high’ phenotype. Sample 3 and 4 represent ‘low’ samples which have both ‘low’-specific haplotypes and ‘low’ phenotype.
(0.39 MB TIF)

Figure S4 Linkage disequilibrium structure for chromosome 7 based on *_Î§_* ^2'^. The linkage disequilibrium coefficient, *_Î§_* ^2'^, indicates that the region between the microsatellite markers, BMS792 and NLBCMS6, harbors strong linkage disequilibrium structure.
(1.36 MB TIF)

Figure S5 Variants of *GRIA1* are observed in both Japanese Black and Holstein. (A) Frequency of S/S (red), S/N (green), and N/N (blue) sires among Japanese Black and Holstein. (B) The average number of AI trials per delivery during two deliveries among daughters derived from S/S, S/N, and N/N fathers in Holstein. AI trials means the number of inseminations needed per pregnancy. Data are presented as mean ± SEM. *P* values were calculated by Student's t-test.
(0.09 MB TIF)

Figure S6 S306N in GRIA1 does not affect expression. (A) The ratio of red (HA, surface expression) and green (GFP, expressed cells) fluorescence intensity in HEK 293 cells expressing GRIA1^Ser^, GRIA1^Asn^, NC, or PC under nonpermeabilizing conditions. Data are presented as mean ± SEM. (B) Representative photos of HEK 293 cells transfected with GRIA1^Ser^, GRIA1^Asn^, NC, or PC under nonpermeabilizing (-Triton) and permeabilizing (+Triton) conditions. (C) Immunoblots of membrane and cytosol fractions extracted from brain of S/S, S/N, and N/N cows.
(0.92 MB TIF)

Figure S7 The concentration of LH in the serum of each animals after PGF treatment. S/S, S/N, and N/N cows were shown in red, green, and blue, respectively.
(0.30 MB TIF)

## References

[1] Hasler JF (1992). Current status and potential of embryo transfer and reproductive technology in dairy cattle.. J Dairy Sci.

[2] Matzuk MM, Lamb DJ (2008). The biology of infertility: research advances and clinical challenges.. Nat Med.

[3] Gonda MG, Arias JA, Shook GE, Kirkpatrick BW (2004). Identification of an ovulation rate QTL in cattle on BTA14 using selective DNA pooling and interval mapping.. Anim Genet.

[4] König S, Bosselmann F, von Borstel UU, Simianer H (2007). Genetic analysis of traits affecting the success of embryo transfer in dairy cattle.. J Dairy Sci.

[5] Dingledine R, Borges K, Bowie D, Traynelis, SF (1999). The glutamate receptor ion channels.. Pharmacol Rev.

[6] Pritchard JK, Stephens MW, Donnelly P (2000). Inference of population structure using multilocus genotype data.. Genetics.

[7] Devlin B, Roeder K, Wasserman L (2001). Genomic control, a new approach to genetic-based association studies.. Theor Popul Biol.

[8] Sved JA (1971). Linkage disequilibrium and homozygosity of chromosome segments in finite populations.. Theor Popul Biol.

[9] Nomura T, Honda T, Mukai F (2001). Inbreeding and effective population size of Japanese Black cattle.. J Anim Sci.

[10] Matsuda S, Yuzaki M (2002). Mutation in hotfoot-4J mice results in retention of δ2 glutamate receptors in ER.. Eur J Neurosci.

[11] Matsuda S, Kamiya Y, Yuzaki M (2005). Roles of the N-terminal domain on the function and quaternary structure of the ionotropic glutamate receptor.. J Biol Chem.

[12] Madry C, Mesic I, Betz H, Laube B (2007). The N-Terminal domains of both NR1 and NR2 subunits determine allosteric Zn^2+^ inhibition and glycine affinity of *N*-methyl-_D_-aspartate receptors.. Mol Pharmacol.

[13] Gielen M, Retchless BS, Mony L, Johnson JW, Paoletti P (2009). Mechanism of differential control of NMDA receptor activity by NR2 subunits.. Nature.

[14] Wetsel WC, Valença MM, Merchenthaler I, Liposits Z, López FJ (1992). Intrinsic pulsatile secretory activity of immortalized luteinizing hormone-releasing hormone-secreting neurons.. Proc Natl Acad Sci USA.

[15] Ojeda SR, Lomniczi A, Mastronardi C, Heger S, Roth C (2006). Minireview: the neuroendocrine regulation of puberty: is the time ripe for a systems biology approach?. Endocrinol.

[16] Schally AV (1970). Hypothalamic regulation of FSH and LH secretion.. Res Reprod.

[17] Fortune JE, Cushman RA, Wahl CM, Kito S (2000). The primordial to primary follicle transition.. Mol Cell Endocrinol.

[18] Felig P, Baxter JD, Broadus AE, Froman LA (1995).

[19] Bevers MM, Dieleman SJ (1987). Superovulation of cows with PMSG: variation in plasma concentrations of progesterone, oestradiol, LH, cortisol, prolactin and PMSG and in number of preovulatory follicles.. Anim Reprod Sci.

[20] Taft R, Ahmad N, Inskeep EK (1996). Exogenous pulses of luteinizing hormone cause persistence of the largest bovine ovarian follicle.. J Anim Sci.

[21] Brann DW, Mahesh VB (1997). Excitatory amino acids: evidence for a role in the control of reproduction and anterior pituitary hormone secretion.. Endocr Rev.

[22] Funahashi T, Hagiwara Y, Kiyu F, Takahashi T (2008). Expression of AMPA receptor subunit glutamate receptor 1 (GluR1) in the preoptic area (POA) delays the onset of puberty in female rats..

[23] Choi JH, Gilks CB, Auersperg N, Leung PC (2006). Immunolocalization of gonadotropin-releasing hormone (GnRH)-I, GnRH-II, and type I GnRH receptor during follicular development in the human ovary.. J Clin Endocrinol Metab.

[24] Ireland JJ, Zielak-Steciwko AE, Jimenez-Krassel F, Folger J, Bettegowda A (2009). Variation in the ovarian reserve is linked to alterations in intrafollicular estradiol production and ovarian biomarkers of follicular differentiation and oocyte quality in cattle.. Biol Reprod.

[25] Zamanillo D, Sprengel R, Hvalby O, Jensen V, Burnashev N (1999). Importance of AMPA receptors for hippocampal synaptic plasticity but not for spatial learning.. Science.

[26] Shimshek DR, Bus T, Grinevich V, Single FN, Mack V (2006). Impaired reproductive behavior by lack of GluR-B containing AMPA receptors but not of NMDA receptors in hypothalamic and septal neurons.. Mol Endocrinol.

[27] Crosignani PG, Walters DE, Soliani A (1991). The ESHRE multicentre trial on the treatment of unexplained infertility: a preliminary report.. Hum Reprod.

[28] Ihara N, Takasuga A, Mizoshita K, Takeda H, Sugimoto M (2004). A comprehensive genetic map of the cattle genome based on 3802 microsatellites.. Genome Res.

[29] Heifetz E, Fulton JE, O'Sullivan N, Zhao H, Dekkers JC (2005). Extent and consistency across generations of linkage disequilibrium in commercial layer chicken breeding populations.. Genetics.

[30] Watanabe T, Hirano T, Takano A, Mizoguchi Y, Sugimoto Y (2008). Linkage disequilibrium structures in cattle and their application to breed identification testing.. Anim Genet.

[31] Wang Y, Matsuda S, Drews V, Torashima T, Meisler MH (2003). A hot spot for hotfoot mutations in the gene encoding the δ2 glutamate receptor.. Eur J Neurosci.

[32] Mikoshiba K, Hatanaka H (1990). Manuals for Neurobiochemistry: Yodosya.

[33] Chen G, Gouaux E (1997). Overexpression of a glutamate receptor (GluR2) ligand binding domain in *Escherichia coli*: application of a novel protein folding screen. Proc.. Natl Acad Sci USA.

[34] Ikeno K, Yamakura T, Yamazaki M, Sakimura K (2001). The Lurcher mutation reveals Ca^2+^ permeability and PKC modification of the GluRδ channels.. Neurosci Res.

[35] Yamazaki M, Ohno-Shosaku T, Fukaya M, Kano M, Watanabe M (2004). A novel action of stargazin as an enhancer of AMPA receptor activity.. Neurosci Res.

[36] Niwa H, Yamamura K, Miyazaki J (1991). Efficient selection for high-expression transfectants with a novel eukaryotic vector.. Gene.

[37] El-Etr M, Akwa Y, Baulieu E, Schumacher M (2006). The neuroactive steroid pregnenolone sulfate stimulates the release of gonadotropin-releasing hormone from GT1-7 hypothalamic neurons, through *N*-methyl-_D_-aspartate receptors.. Endocrinol.

[38] Kengaku K, Tanaka T, Kamomae H (2007). Changes in the peripheral concentrations of inhibin, follicle-stimulating hormone, luteinizing hormone, progesterone and estradiol-17β during turnover of cystic follicles in dairy cows with spontaneous follicular cysts.. J Reprod Dev.

[39] Mori Y, Kano Y (1984). Changes in plasma concentrations of LH, progesterone and oestradiol in relation to the occurrence of luteolysis, oestrus and time of ovulation in the Shiba goat (Capra hircus).. J Reprod Fertil.

