# Supplementary figures and images for: Ionotropic Glutamate Receptor AMPA 1 Is Associated with Ovulation Rate
Source: PLoS One. 2010 Nov 3;5(11):e13817. doi: 10.1371/journal.pone.0013817 (PMC2972219; doi:10.1371/journal.pone.0013817)

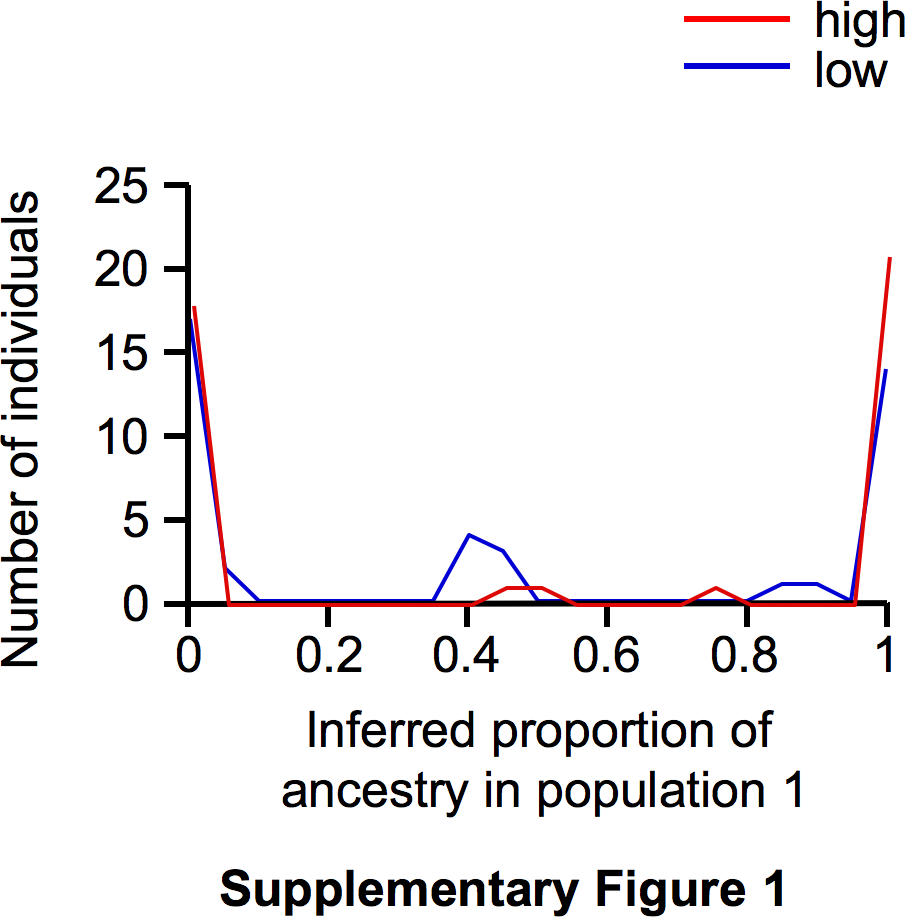

Supplement: Figure S1 — The population structure of analyzed samples based on STRUCTURE. The population structure of our samples was estimated with STRUCTURE [6].155 markers were extracted from 1154 markers, with at least a 20-cM interval. We set 100,000 Markov chain Monte Carlo interactions including 10,000 burn-in interactions, and assumed the subpopulation number to be 2. Eighty-four individuals were separated into populations 1 and 2. The inferred proportion of ancestry in population 1 of high (red) and low (blue) were similar. (0.06 MB TIF) [file pone.0013817.s001.tif]

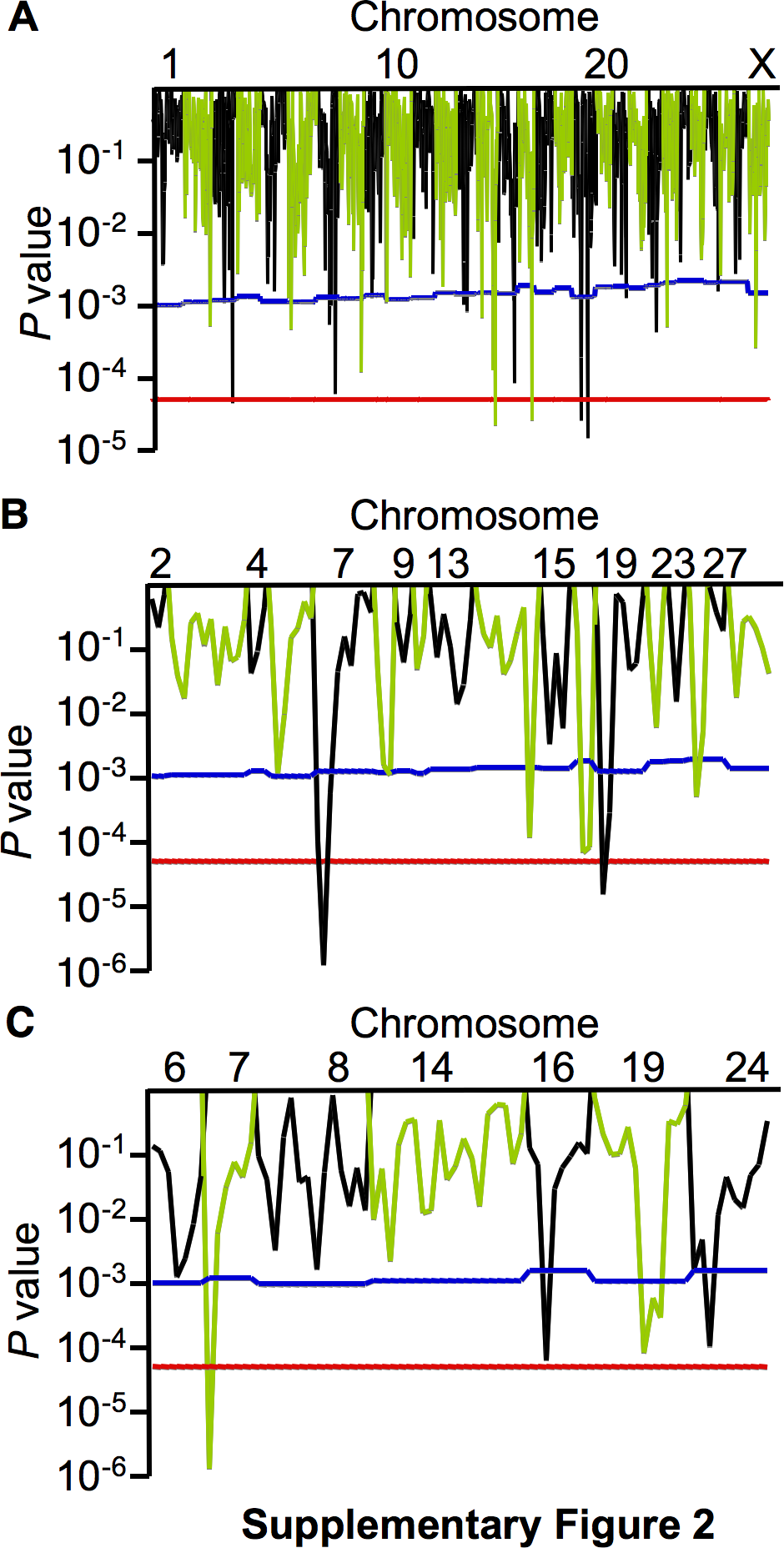

Supplement: Figure S2 — Chromosome 7 is associated with ovulation rate in cattle. (A–C) Association signals with ovulation rate using plots of the P values for Fisher's exact test after estimating the haplotypes of consecutive marker pairs by the expectation-maximization algorithm. Different bands of green are used to differentiate marker pairs on consecutive chromosomes. Blue and red lines represent the thresholds for chromosome-wise and genome-wise significance based on Bonferroni's correction for multiple comparisons, respectively. (A) Genome-wide scans. (B) Scans at selected chromosomes with additional samples. (C) Scans at selected chromosomes with additional markers. (0.39 MB TIF) [file pone.0013817.s002.tif]

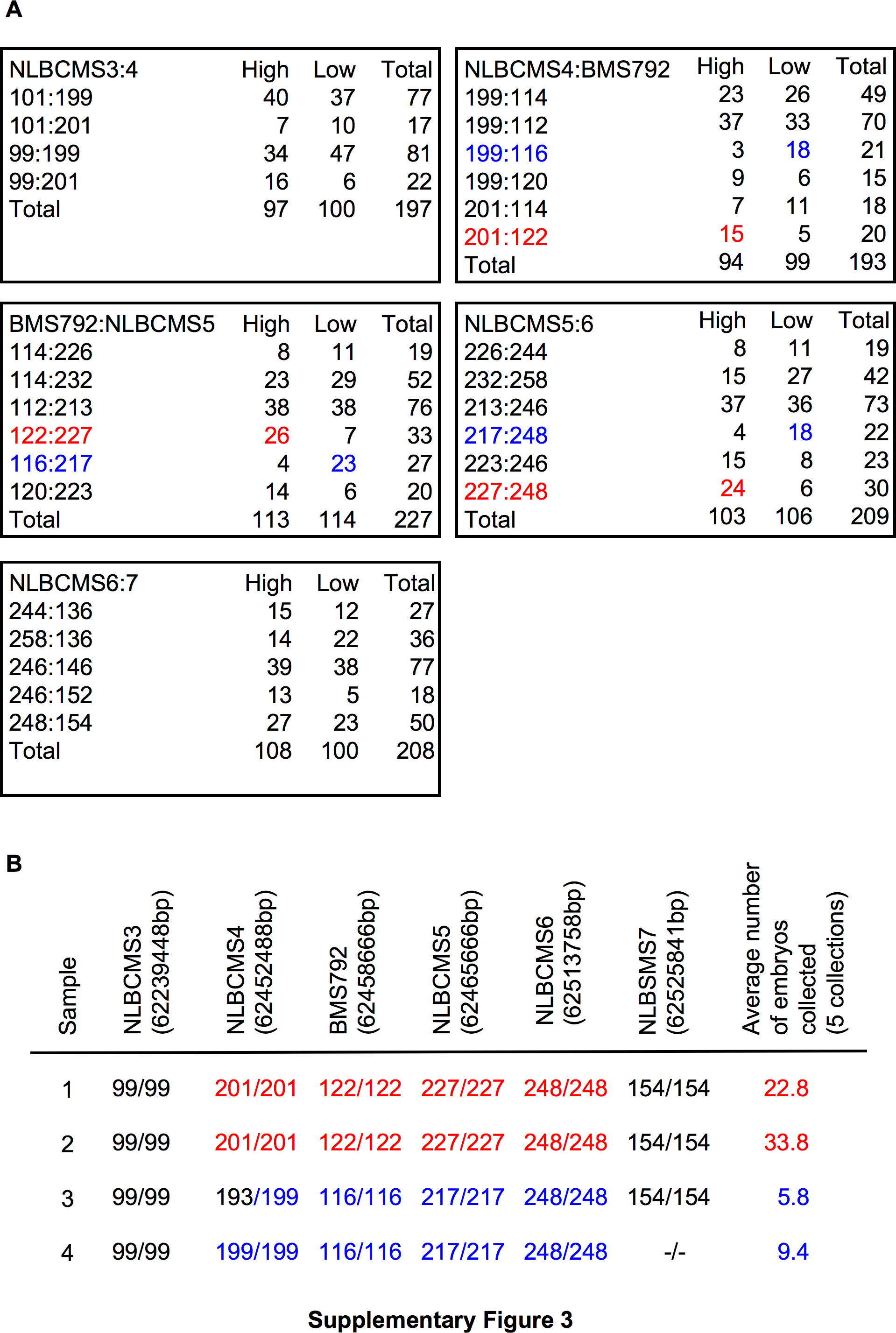

Supplement: Figure S3 — Samples for sequencing are selected by their haplotypes. (A) Frequency of haplotypes of consecutive marker pairs in the candidate region. Red and blue indicate ‘high’- and ‘low’-specific haplotypes, respectively. NLBCMS3, 4, 5, 6, and 7 and BMS792 are microsatellite markers located in the critical region. (B) Selected samples for sequencing. Sample 1 and 2 represent ‘high’ samples which have both ‘high’-specific haplotypes and ‘high’ phenotype. Sample 3 and 4 represent ‘low’ samples which have both ‘low’-specific haplotypes and ‘low’ phenotype. (0.39 MB TIF) [file pone.0013817.s003.tif]

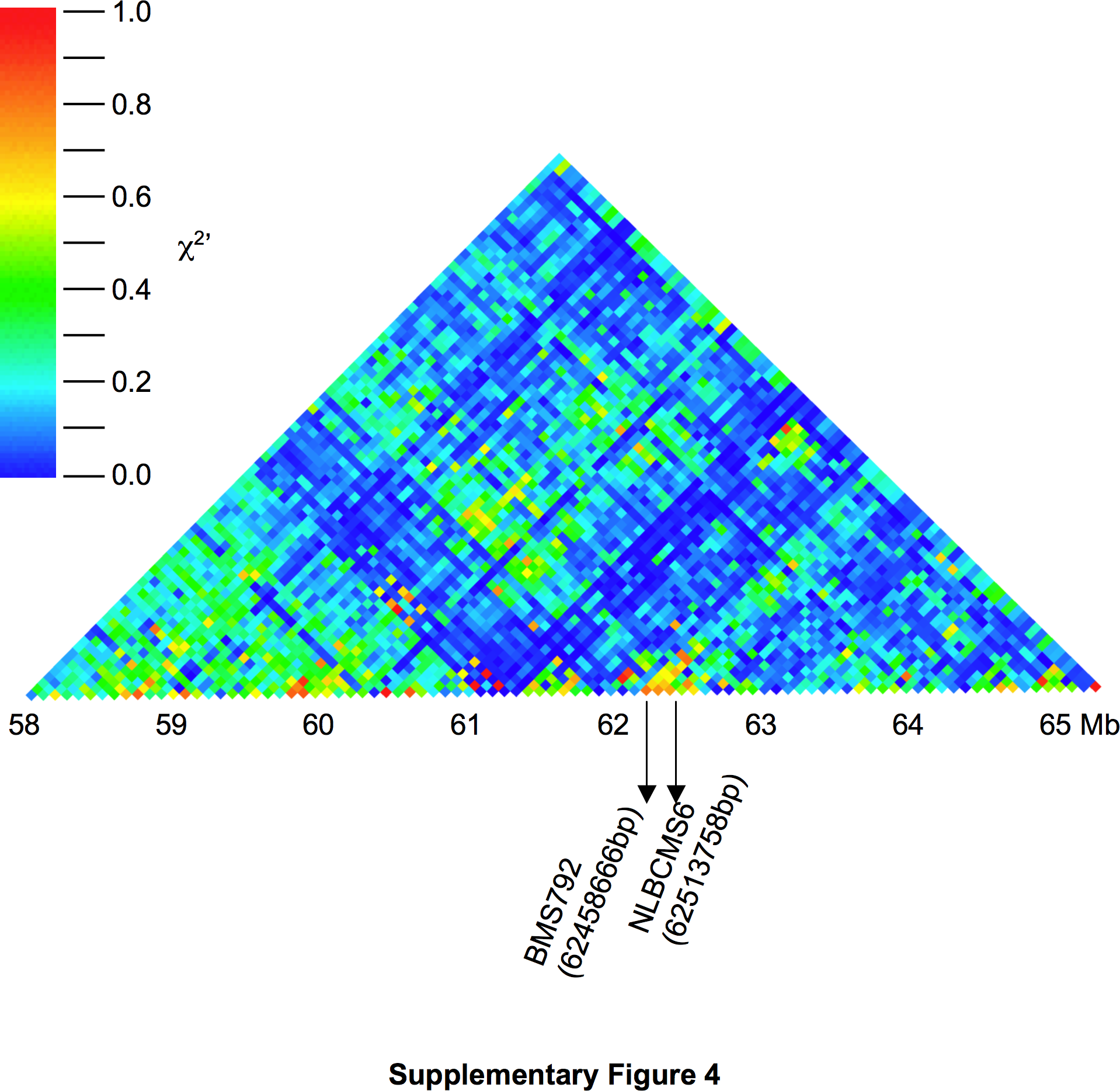

Supplement: Figure S4 — Linkage disequilibrium structure for chromosome 7 based on Î§ 2'. The linkage disequilibrium coefficient, Î§ 2', indicates that the region between the microsatellite markers, BMS792 and NLBCMS6, harbors strong linkage disequilibrium structure. (1.36 MB TIF) [file pone.0013817.s004.tif]

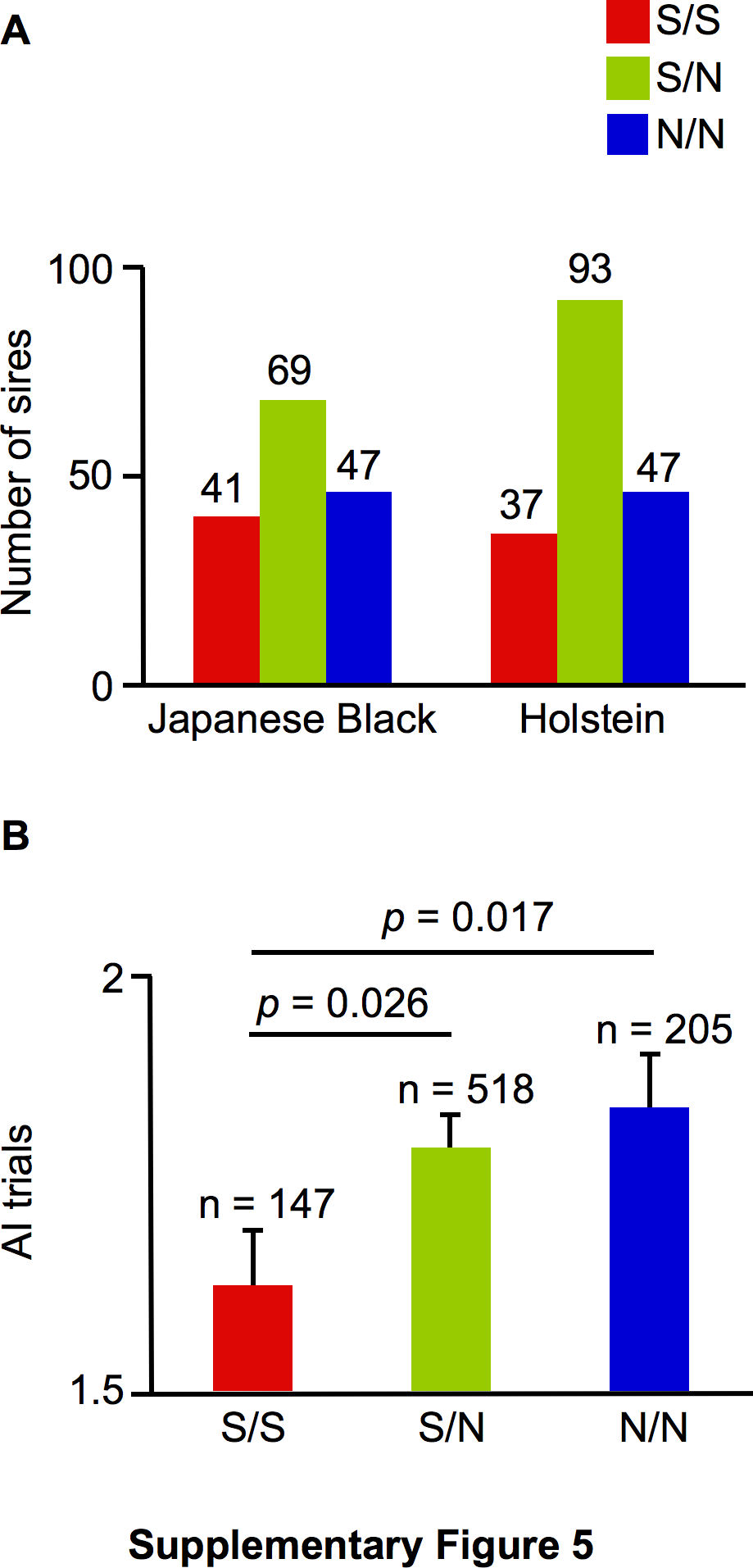

Supplement: Figure S5 — Variants of GRIA1 are observed in both Japanese Black and Holstein. (A) Frequency of S/S (red), S/N (green), and N/N (blue) sires among Japanese Black and Holstein. (B) The average number of AI trials per delivery during two deliveries among daughters derived from S/S, S/N, and N/N fathers in Holstein. AI trials means the number of inseminations needed per pregnancy. Data are presented as mean ± SEM. P values were calculated by Student's t-test. (0.09 MB TIF) [file pone.0013817.s005.tif]

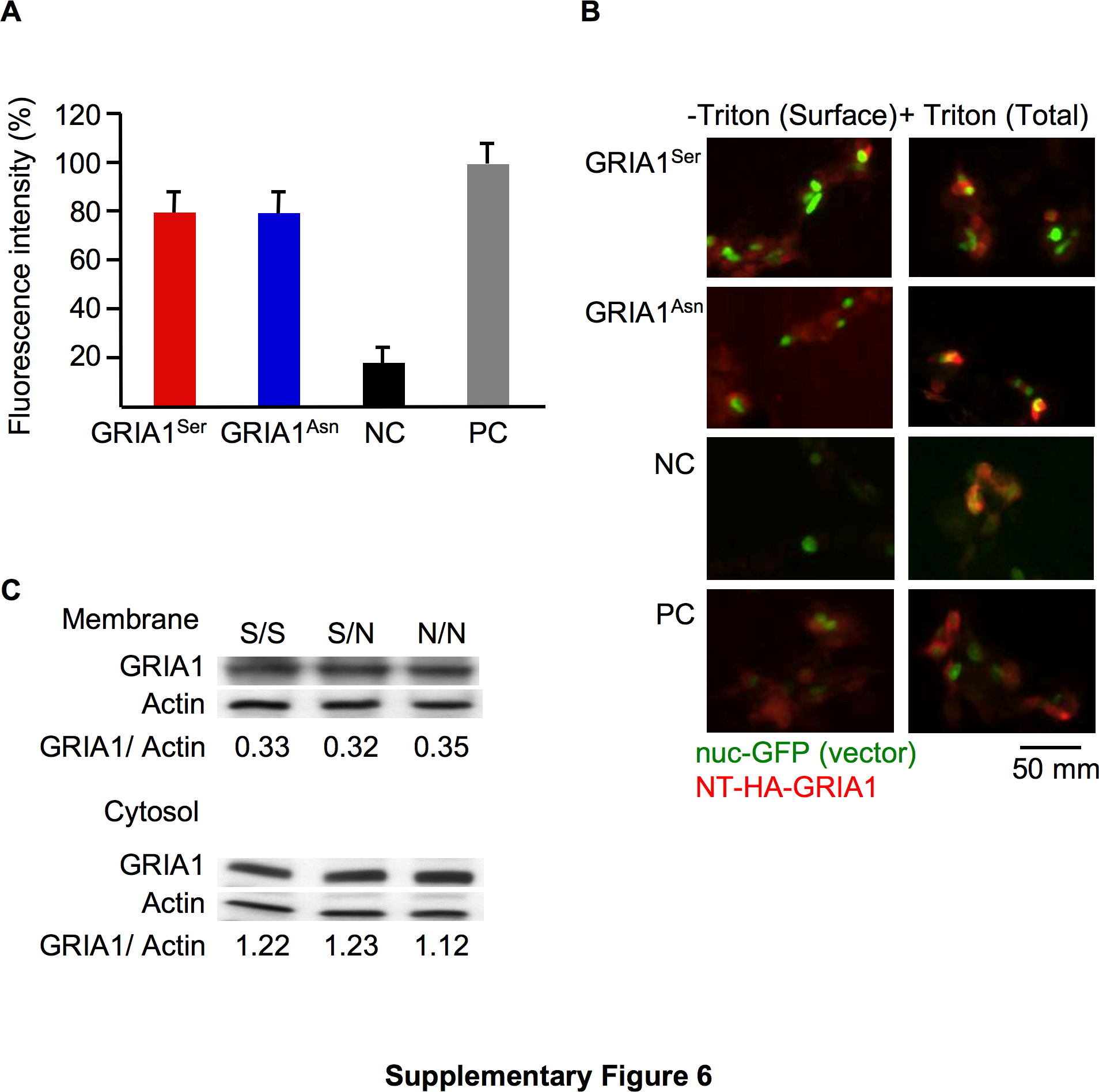

Supplement: Figure S6 — S306N in GRIA1 does not affect expression. (A) The ratio of red (HA, surface expression) and green (GFP, expressed cells) fluorescence intensity in HEK 293 cells expressing GRIA1Ser, GRIA1Asn, NC, or PC under nonpermeabilizing conditions. Data are presented as mean ± SEM. (B) Representative photos of HEK 293 cells transfected with GRIA1Ser, GRIA1Asn, NC, or PC under nonpermeabilizing (-Triton) and permeabilizing (+Triton) conditions. (C) Immunoblots of membrane and cytosol fractions extracted from brain of S/S, S/N, and N/N cows. (0.92 MB TIF) [file pone.0013817.s006.tif]

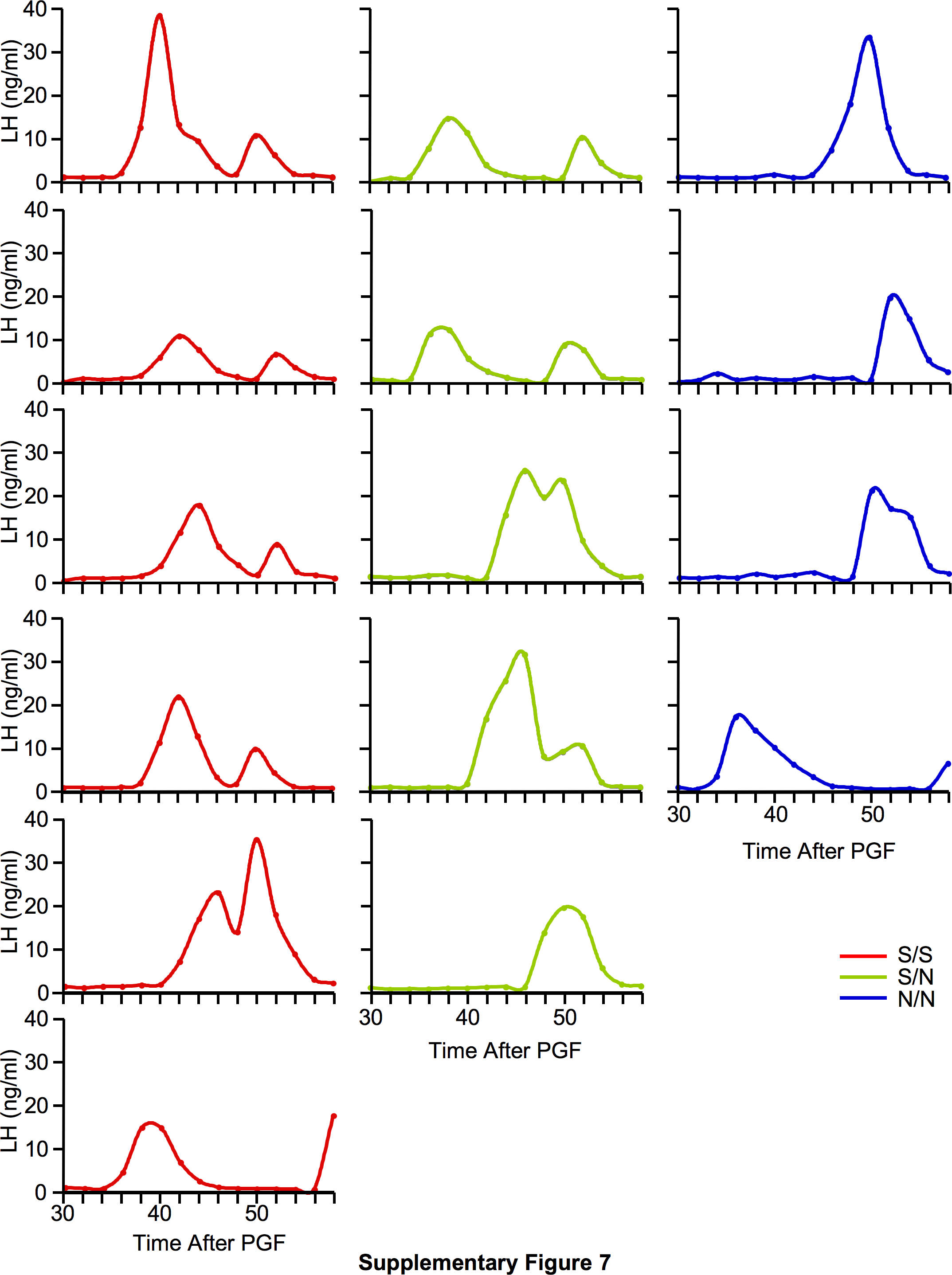

Supplement: Figure S7 — The concentration of LH in the serum of each animals after PGF treatment. S/S, S/N, and N/N cows were shown in red, green, and blue, respectively. (0.30 MB TIF) [file pone.0013817.s007.tif]
